# Recombinant Production of MFHR1, A Novel Synthetic Multitarget Complement Inhibitor, in Moss Bioreactors

**DOI:** 10.3389/fpls.2019.00260

**Published:** 2019-03-20

**Authors:** Oguz Top, Juliana Parsons, Lennard L. Bohlender, Stefan Michelfelder, Phillipp Kopp, Christian Busch-Steenberg, Sebastian N. W. Hoernstein, Peter F. Zipfel, Karsten Häffner, Ralf Reski, Eva L. Decker

**Affiliations:** ^1^ Department of Plant Biotechnology, Faculty of Biology, University of Freiburg, Freiburg, Germany; ^2^ Spemann Graduate School of Biology and Medicine (SGBM), University of Freiburg, Freiburg, Germany; ^3^ Faculty of Medicine, Department of General Pediatrics, Adolescent Medicine and Neonatology, Medical Center - University Freiburg, University of Freiburg, Freiburg, Germany; ^4^ Leibniz Institute for Natural Product Research and Infection Biology, Friedrich Schiller University, Jena, Germany; ^5^ Signalling Research Centres BIOSS and CIBSS, University of Freiburg, Freiburg, Germany

**Keywords:** *Physcomitrella patens*, moss bioreactor, factor H, plant-made recombinant pharmaceuticals, synthetic complement inhibitor, alternative pathway of complement activation, aHUS, C3 glomerulopathy

## Abstract

The human complement system is an important part of the immune system responsible for lysis and elimination of invading microorganisms and apoptotic body cells. Improper activation of the system due to deficiency, mutations, or autoantibodies of complement regulators, mainly factor H (FH) and FH-related proteins (FHRs), causes severe kidney and eye diseases. However, there is no recombinant FH therapeutic available on the market. The first successful recombinant production of FH was accomplished with the moss bioreactor, *Physcomitrella patens*. Recently, a synthetic regulator, MFHR1, was designed to generate a multitarget complement inhibitor that combines the activities of FH and the FH-related protein 1 (FHR1). The potential of MFHR1 was demonstrated in a proof-of-concept study with transiently transfected insect cells. Here, we present the stable production of recombinant glyco-engineered MFHR1 in the moss bioreactor. The key features of this system are precise genome engineering *via* homologous recombination, Good Manufacturing Practice-compliant production in photobioreactors, high batch-to-batch reproducibility, and product stability. Several potential biopharmaceuticals are being produced in this system. In some cases, these are even biobetters, i.e., the recombinant proteins produced in moss have a superior quality compared to their counterparts from mammalian systems as for example moss-made aGal, which successfully passed phase I clinical trials. *Via* mass spectrometry-based analysis of moss-produced MFHR1, we now prove the correct synthesis and modification of this glycoprotein with predominantly complex-type N-glycan attachment. Moss-produced MFHR1 exhibits cofactor and decay acceleration activities comparable to FH, and its mechanism of action on multiple levels within the alternative pathway of complement activation led to a strong inhibitory activity on the whole alternative pathway, which was higher than with the physiological regulator FH.

## Introduction

Biopharmaceutical production is a steadily growing field within the pharmaceutical market (International Federation of Pharmaceutical Manufacturers & Associations IFPMA, 2017). Plant-based systems can offer advantages in sectors neglected by the mainstream bacterial or mammalian systems and their current business models (Stoger et al., 2014). Impressive recent examples for fully developed plant production processes comprise, e.g., Protalix, the carrot-cell-derived Taliglucerase alfa from a stable production process, as well as the transiently expressed ZMapp antibody cocktail to fight Ebola infections (Zimran et al., 2011; Lomonossoff and D’Aoust, 2016), the announcement of the clinical phase 3 start for the seasonal quadrivalent influenza vaccine produced in *Nicotiana benthamiana*^1^ and the successful completion of clinical phase 1 for recombinant alpha-galactosidase A (moss-aGal) produced in *Physcomitrella patens*^2^.

The development of pharmaceuticals treating diseases, which are associated with malfunction of the human complement system, may be an attractive field for plant-based production. As part of the innate immune system, the complement system is a crucial defense line against invading microorganisms (Thurman and Holers, 2006). Furthermore, it is essential for tissue homeostasis by discriminating damaged or apoptotic host cells from healthy tissues and promoting their elimination (Janeway and Medzhitov, 2002; Zipfel and Skerka, 2009). The activity of more than 50 plasma proteins functioning in a cascade of enzymatic reactions, either in plasma (fluid-phase) or on cell surfaces, has to be tightly controlled by regulatory proteins. The malfunction of these complement regulators can lead to over-activation of the system with the consequence of severe diseases, especially of the kidney and eyes (Józsi and Zipfel, 2008).

**Figure 1:**
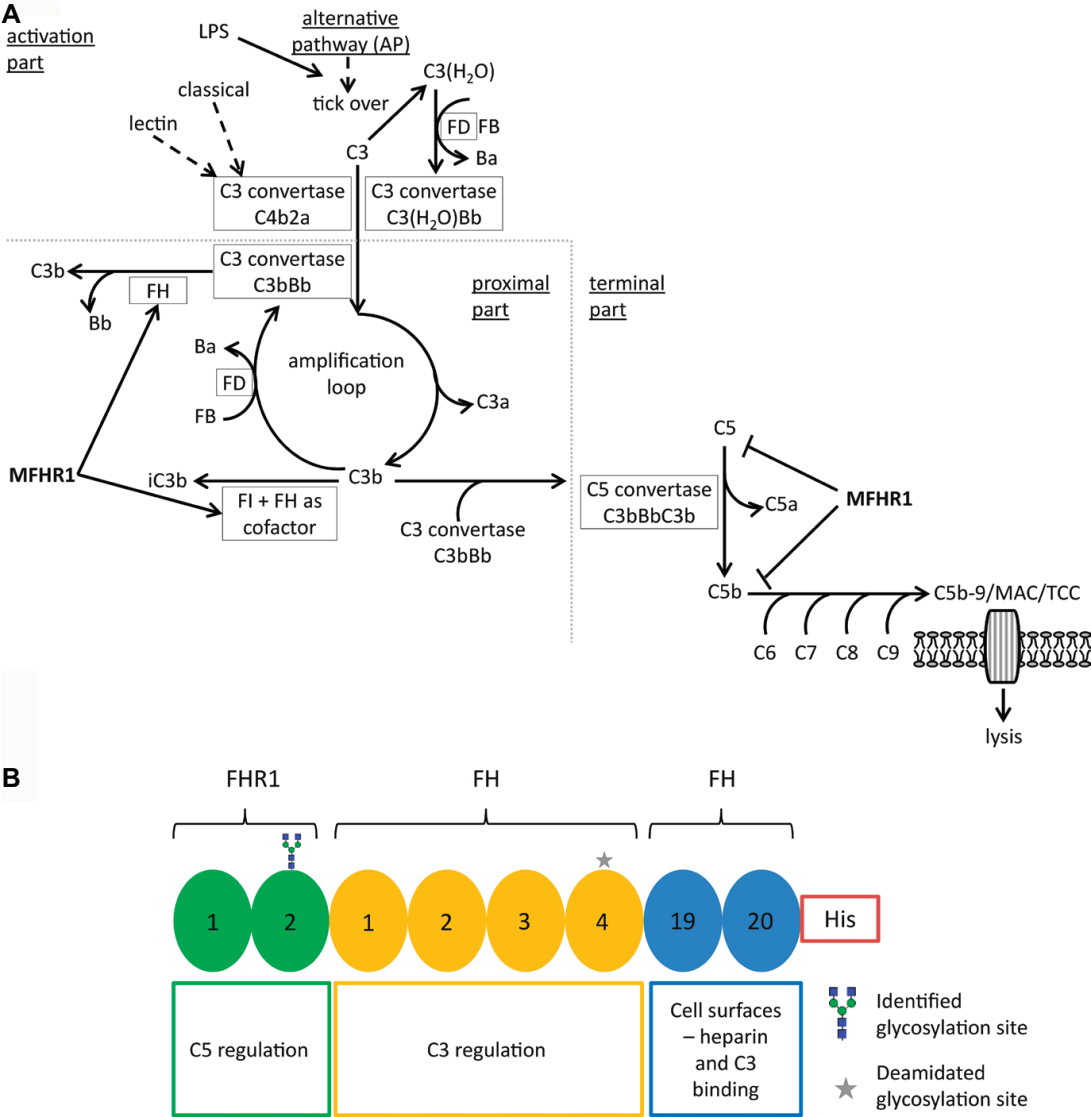
Schematic representation of MFHR1 activity mechanism and structure. **(A)** MFHR1 regulates the alternative pathway simultaneously at different stages. Processes promoted by MFHR1 or activated by lipopolysaccharide (LPS) are marked by arrows and the step inhibited by MFHR1 is marked by a blocking arrow. F: complement regulatory factor, C: complement component, a or b: cleavage fragments, iC3b: inhibited C3b, AP: alternative pathway, LPS: lipopolysaccharide, MAC: membrane attack complex, TCC: terminal complement complex. **(B)** The synthetic MFHR1 protein comprises eight short consensus repeats (SCR), namely FHR1_1-2_, FH_1-4_ and FH_19-20_, without artificial linkers and an 8x histidine tag at the C-terminus (His).

The complement system can be activated by three different mechanisms, the classical, lectin, and alternative pathway. They merge at the level of C3 activation by generating variants of the so-called C3 convertase, an enzymatic complex able to cleave C3 molecules into the active forms C3b, an opsonin, and C3a, an anaphylatoxin (Figure 1A; Thurman and Holers, 2006). The alternative pathway of complement activation (AP) constitutively hydrolyzes C3 at low levels, generating C3(H_2_O), and subsequently C3(H_2_O)Bb, the fluid-phase C3 convertase. In the so-called amplification loop, the C3 convertase produces more C3b molecules, and additional C3 convertase (C3bBb) is formed (Zipfel and Skerka, 2009). This pathway can also be initiated by the presence of bacterial lipopolysaccharides (LPS) (Pangburn et al., 1980). The last step in the proximal part of the alternative pathway comprises the addition of another C3b molecule to the C3 convertase, thus forming the C5 convertase (C3bBbC3b). The terminal pathway, common to all three routes of complement activation, starts with the C5 convertase-catalyzed cleavage of C5 to C5a and C5b and involves the non-enzymatic assembly of a complex of C5b with the plasma proteins C6, C7, C8, and C9. This complex, the membrane attack complex (MAC) or terminal complement complex (TCC), creates a pore on cell membranes leading to the lysis of target cells (Figure 1A; Morgan et al., 2016).

As C3b binds to infectious microbes as well as adjacent host cells alike and marks them for lysis, host tissues have to be protected from damage with the help of complement regulatory proteins (De Córdoba and De Jorge, 2008). Factor H (FH) is the main regulator of the activation and amplification of the alternative pathway cascade. It is a single-chain plasma glycoprotein consisting of 20 globular short consensus repeat (SCR) domains (Zipfel et al., 1999) and is active in the fluid phase as well as on host cell surfaces. FH inhibits C3-convertase (C3bBb) formation by competing with FB for binding to C3b and promotes the irreversible dissociation of preformed C3bBb (decay acceleration activity) (Hourcade et al., 1999). It also acts as a cofactor for the factor I (FI)-dependent inactivation of C3b in the fluid phase, the so-called cofactor activity of FH. While these activities reside in the N-terminal SCRs 1–4 of FH (Kühn et al., 1995), the C-terminal SCRs 19–20 interact with cell surfaces *via* binding to polyanions, such as glycosaminoglycans (GAGs), thus protecting host cells from complement attack.

Factor H forms a small family with five related proteins (FHR1–5), also composed of SCR domains which share a high degree of sequence identity (Skerka et al., 2013). FHR1 regulates the terminal complement pathway by binding to C5, preventing its activation and inhibiting TCC assembly later in the cascade (Heinen et al., 2009). In addition, homo- or heterodimers of FHR1, FHR2, and FHR5 can compete with FH for binding to polyanions resulting in a decrease of FH levels with the consequence of local complement activation on host cell surfaces (Fritsche et al., 2010). Although the exact role of FHRs on complement regulation is not yet fully clarified, it is proposed that expression levels and ratios of the different FH-family members are necessary for fine-tuning of complement regulation (Józsi and Zipfel, 2008; Goicoechea de Jorge et al., 2013; Skerka et al., 2013).

Mutations in FH, mainly in the carboxy-terminus of the protein can lead to an ineffective local regulation of the complement system on host cells causing damage of tissues, especially on endothelia, and lead to microangiopathic hemolytic anemia and acute renal failure known as atypical hemolytic uremic syndrome (aHUS) (Józsi et al., 2005). Autoantibodies against FH or FH deficiency or mutations may cause an over-activation of the complement cascade and uncontrolled cleavage of C3, followed by a depletion of plasma C3 and accumulation of C3-cleavage products on the glomerular basement membrane of the kidney. These depositions are typical in C3 glomerulopathies (C3G) and lead to renal failure (Pickering et al., 2002; Noris and Remuzzi, 2015). Age-related macular degeneration (AMD), the major cause of irreversible loss of central vision, especially in the elderly population, is also linked to genetic variants of complement components, among others FH (Fritsche et al., 2016; Geerlings et al., 2017).

Treatment options for complement-associated renal diseases are limited. FH-substitution *via* plasmapheresis was shown to restore normal complement activity in aHUS and C3G patients (Cataland and Wu, 2014). The use of Eculizumab, a monoclonal antibody inhibiting C5 activation and one of the most expensive pharmaceuticals worldwide, has significantly improved the clinical treatment of aHUS and PNH patients (Wong and Kavanagh, 2015). However, Eculizumab could not prevent the activation of C5 sufficiently for every patient (Harder et al., 2017). Moreover, Eculizumab is not effective in many patients suffering from C3G because it does not act on C3 level, thus does not prevent the accumulation of C3 cleavage products (Bomback et al., 2012). For these patients, the use of the physiological regulator FH will be beneficial, as it already acts on the level of C3 activation and inhibits over-activation of the system locally on host cells. In addition, the side effects of systemic inhibition treatment, e.g. a higher risk of infections (Fridkis-Hareli et al., 2011), will be avoided.

Recombinant FH was already successfully produced in *P. patens*. Moss-produced FH (mossFH) showed full *in vitro* complement regulatory activity, and it efficiently blocked AP activation and hemolysis induced by sera from aHUS patients (Büttner-Mainik et al., 2011; Michelfelder et al., 2017). Moreover, in a preclinical study, it decreased the pathological deposition of C3 cleavage products and increased the levels of plasma C3 in a murine C3G model (Michelfelder et al., 2017). Recombinant proteins modulating the complement system on multiple levels of the activation cascade might be the key to the development of new therapeutics to treat complement-related disorders. This strategy aims at the generation of smaller proteins with higher activity, implying that lower amounts of protein would be necessary to achieve the desired therapeutic effect. This is desirable, not only from the production point of view but also because of higher convenience for the patient. Recently, a novel synthetic multitarget regulator, MFHR1, was designed to combine the terminal pathway-regulating and dimerization domains of FHR1 with the functionally relevant C3-regulating and surface-binding domains of FH (Figure 1B; Michelfelder et al., 2018). After demonstrating the potential of MFHR1 as a biopharmaceutical in a proof-of-concept study using protein transiently expressed in insect cells, we now aimed to establish a stable production process for MFHR1 in the moss (*P. patens*) bioreactor. The moss *P. patens* is an important model species in basic research as well as a biotechnology production platform with outstanding features including a fully sequenced genome, efficient homologous recombination-based genome-engineering, dominant haploid gametophytic stage in life cycle and Good Manufacturing Practice (GMP)-compliant production in moss photobioreactors (Rensing et al., 2008; Decker and Reski, 2012; Decker et al., 2014; Reski et al., 2015; Lang et al., 2018; Wiedemann et al., 2018). In the end of 2017, a milestone was reached when Greenovation Biotech GmbH successfully completed the phase I clinical study for the first moss-produced drug candidate, moss-aGal, against Fabry disease (Reski et al., 2018). Moreover, mossFH is currently being tested in preclinical trials and showed that it successfully reduced C3 deposits in a FH-deficient mouse model (Häffner et al., 2017; Michelfelder et al., 2017).

In this work, we report the successful stable production of the novel synthetic multitarget complement inhibitor MFHR1, a potentially promising pharmaceutical product, in the GMP-compliant production platform *P. patens*. Moss-produced MFHR1 was fully characterized by mass spectrometry. It retains the regulatory activity from both originating proteins, FH and FHR1, and displays a higher inhibitory activity *in vitro* on the whole alternative pathway than FH, thus being a promising future biopharmaceutical product to cure complement-associated diseases.

## Results

### Transgenic Plant Generation

For the production of MFHR1, the *∆xt/ft* moss parental line was used, a double knockout for the α1,3 fucosyltransferase and the β1,2 xylosyltransferase genes, which has been generated by gene targeting *via* homologous recombination and has been used previously for the production of FH (Michelfelder et al., 2017). This plant generates N-glycans without α1,3-attached fucose or β1,2-attached xylose (Koprivova et al., 2004; Huether et al., 2005; Michelfelder et al., 2017). MFHR1 expression was driven by the PpActin5 promoter (Weise et al., 2006) and targeting to the secretory pathway for its proper posttranslational modification was achieved by using the aspartic proteinase signal peptide of PpAP1 (Schaaf et al., 2004, 2005).

Plants surviving the selection procedure were directly screened by PCR for the presence of the transgene in the moss genome (Supplementary Figure 1) and *via* a sandwich ELISA for the production of MFHR1, from extracts of plant material grown on solid medium. Plants with a positive signal were transferred to liquid culture and screened again for productivity *via* ELISA (Supplementary Table 1). The two best-producing clones, P1 and P5, were selected for photobioreactor cultivation. Line P5 showed a slower biomass increase (Supplementary Figure 2). Therefore, according to its wild-type-like growth behavior and the overall level of MFHR1 production, P1 was chosen for further experiments.

### Establishment of a Production and Purification Process for mossMFHR1

**Figure 2:**
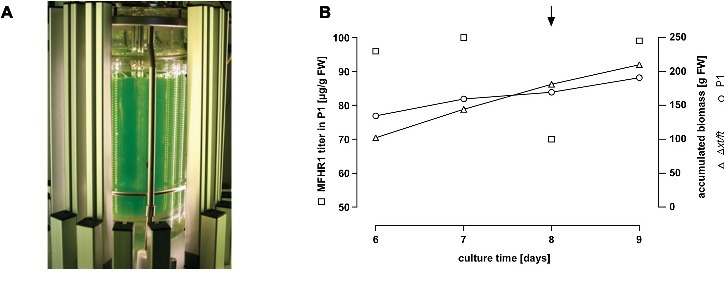
MFHR1 production in the moss bioreactor. **(A)** Five liter photobioreactor setup of a *Physcomitrella patens* culture illuminated with LED lamps (in-house design). **(B)** Kinetics of MFHR1 concentration and biomass accumulation of the parental line Δ*xt/ft* and P1 in a culture carried out in the photobioreactor. The arrow marks the addition of 5 μM NAA to the culture.

In order to accumulate biomass for the purification of MFHR1, the process in the photobioreactor was executed as a batch without harvesting material for 6 days (Figure 2A). From this time point onward, 2 L of suspension were harvested every day and replaced by fresh medium. The amount of biomass harvested in this 9-day process attained 190 g fresh weight (FW), and the growth index was 53.4 (GI = (biomass_final_−biomass_initial_)/biomass_initial_). Under these conditions, the concentration of MFHR1 in the plant material reached 100 μg MFHR1/g FW and was constant until day 8, when it started to decrease (Figure 2B). Previous experiments have shown that the concentration of the protein of interest decreased further with the time of culture (Supplementary Figure 2). In the culture conditions used, the addition of 5 μM naphthaleneacetic acid (NAA), a synthetic auxin, increased intracellular MFHR1 concentrations until the whole plant material was harvested 1 day later. All in all, more than 17 mg MFHR1 accumulated in the 5 L bioreactor within 9 days.

**Figure 3:**
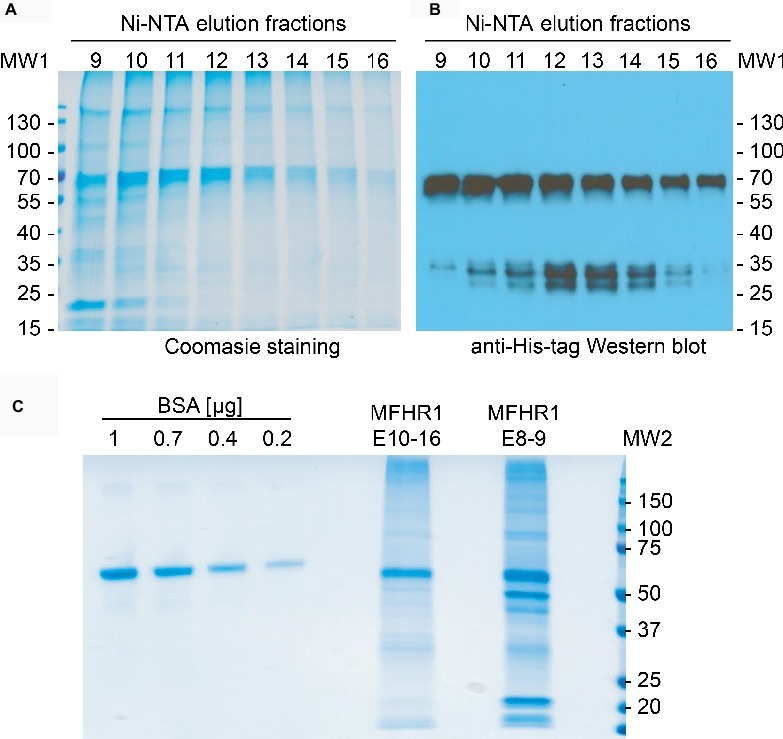
Purification of mossMFHR1. **(A)** Coomassie staining of MFHR1 elution fractions 8-13, purified *via* Ni-NTA chromatography. **(B)** Western blot of the same elution fractions detected with anti-His-tag antibodies. MW1: PageRuler Prestained Protein Ladder (Thermo Fisher Scientific). **(C)** Coomassie staining of MFHR1 elution fraction pools (E8-9 and E10-16) and BSA as a reference. MW2: Precision Plus Protein™ Standard (BioRad).

His-tagged MFHR1 was extracted from 6- to 9-day-old plant material and purified *via* Ni-NTA chromatography. As measured *via* ELISA, the protein of interest eluted at an imidazole concentration above 250 mM, between fraction 6 and 16 (Supplementary Figure 3). Additionally, Coomassie staining and Western blotting confirmed the signal at the expected size of 58 kDa (Figures 3A,B). Moreover, fractions 10–16 displayed suitable purity of the protein of interest (Figure 3A). A small amount of degraded protein was observed. The elution fractions 10–16 were collected, dialyzed against DPBS, concentrated *via* membrane ultrafiltration, and used for activity tests. Concentrated elution fractions 10–16 from homogenates of the parental plant *Δxt/ft* were used as negative control for any activity of moss-endogenous proteins. In both cases, fractions up to fraction 9 were discarded due to host-cell protein contamination as assessed by SDS-PAGE and Coomassie staining (Figure 3C). As MFHR1 is a novel fusion protein, a protein standard for quantification *via* ELISA had to be produced. After purification *via* Ni-NTA and ultrafiltration, the concentration of the protein of interest was assessed by band densitometry on Coomassie-stained gels, using BSA as a standard for protein amount (Figure 3C, E10–16). The concentration of mossMFHR1 used for activity assays relies on ELISA-quantifications using this standard.

### Structural Analyses Prove Correct Synthesis and Complex-Type N-Glycosylation of mossMFHR1

**Figure 4:**
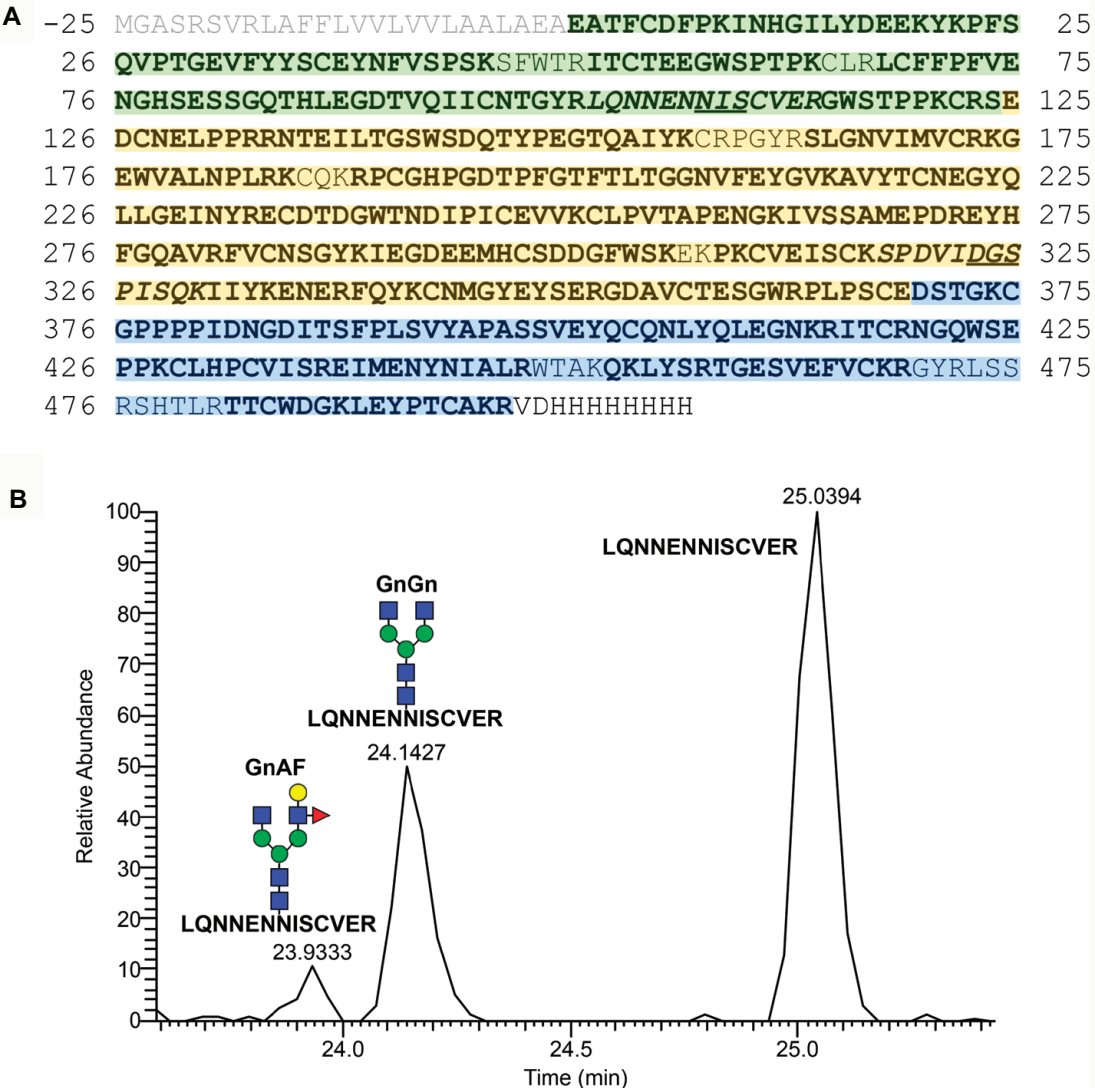
Sequence and domain structure of MFHR1 and mass spectrometric sequence and N-glycosylation analyses. **(A)** Mature MFHR1 sequence (black) fused to the AP1-signal peptide (grey). The amino acid positions relating to the mature protein are given. Negative numbers refer to the signal peptide. The amino-terminal portion of MFHR1 is composed of the first two SCRs of FHR1 (green), followed by the catalytically active SCRs 1-4 from FH (yellow). The C-terminus includes the surface-binding SCRs 19-20 from FH (blue) followed by a His-tag. Peptides identified by mass spectrometry are shown in bold and the putative N-glycosylation site NIS (Asn108) and the deamidated site DGS (Asn323 ➔ Asp323), are underlined, corresponding mass spectrometric detected tryptic peptides are shown in italics. **(B)** Elution profiles (extracted ion chromatogram, EIC) of the tryptic peptide (_102_LQNNENNISCVER_113_) which flanks the glycosylation site NIS (Asn108) with and without N-glycosylation. Peak identity was confirmed by m/z-value and charge state on MS1- and by reporter ions on MS2-level (see Supplementary Figure 4 for further information). Peak quantification revealed an N-glycan occupancy at Asn108 of 35%, which in total consisted of 86% GnGn−, nearly 14% GnAF structures and < 1% of N-glycans with terminal mannoses, all of them lacking plant specific core xylose and fucose. For the deamidated site (Asn323 ➔ Asp323) no N-glycosylation was detectable. Gn: N-acetylglucosamine, A: galactose, F: Fucose – referring to the terminal sugar residues.

Purified mossMFHR1 was identified with a sequence coverage of 91% by mass-spectrometric analysis (Figure 4). We can assume the correct cleavage of the signal peptide, as this peptide was not detected in the MS analysis, and the N-terminus of the mature protein could be confirmed. The carboxy-terminal His-tag remains undetected due to the low mass/charge ratio of this peptide, but nevertheless it is present, as MFHR1 was purified *via* Ni-NTA affinity chromatography. Furthermore, we analyzed the N-glycosylation of mossMFHR1. As previously reported for both, human-derived and mossFH (Fenaille et al., 2007; Michelfelder et al., 2017), the putative glycosylation site NGS, originated from SCR4 of FH and located in SCR6 from MFHR1, was found to be deamidated to DGS, and therefore not glycosylated. Besides, the glycosylation site NIS located in SCR2 and derived from FHR1 was occupied with glycans in only 35% of the peptides, while 65% were not modified (Figure 4B, Supplementary Figure 4). This is in agreement with the situation of FHR1 derived from human plasma where two different isoforms, FHR1α and FHR1β, 37 and 42 kDa, with one and two attached carbohydrate chains respectively, occur (Skerka and Zipfel, 2008). Approximately 86% of the glycosylated MFHR1 displayed the complex type N-glycan GnGn. Structures exhibiting terminal mannoses were below 1%. Peptides with N-glycans bearing β1,2-linked xylose and α1,3-linked fucose were not detected. The lack of β1,2-linked xylose and α1,3-linked fucose residues had been shown before for the parental line *Δxt/ft* (Michelfelder et al., 2017). N-glycans decorated with Lewis A structures, the trisaccharide Fucα1–4(Galβ1–3)GlcNAc (Parsons et al., 2012), were detected in up to 14% of the product and on only one antenna of the sugar tree (GnAF). To sum up, moss-produced MFHR1 was complete and complex-type N-glycosylated.

### MossMFHR1 Displays Cofactor Activity as Early Regulatory Function in Complement Activation

**Figure 5:**
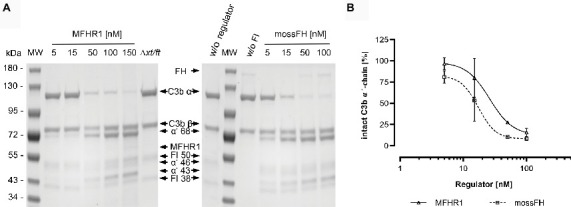
The synthetic moss-produced complement regulator MFHR1 displays cofactor activity. **(A)** MossMFHR1 and mossFH act as cofactors for the FI-dependent cleavage of C3b in the fluid phase in a dose-dependent manner. The cleavage products were detected after SDS-PAGE and Coomassie staining. MW: PageRuler Prestained Protein Ladder (Thermo Fisher Scientific); w/o regulator: no addition of MFHR1 or FH; w/o FI: no addition of FI; *Δxt/ft*: parental line, negative control. **(B)** MossMFHR1 shows similar cofactor activity compared to mossFH (*p* > 0.05, two-way ANOVA). The control without any regulator was set to 100%. Data represent mean values and ± SD from three independent experiments. Some error bars are shorter than the symbol. Analyses were done with GraphPad Prism software version 8.0 for Windows.

The first activity of FH within the regulation of the alternative complement pathway is its role as cofactor for FI-mediated cleavage of C3b in the fluid phase (cofactor activity of FH; Zipfel et al., 1999). To assess whether this important function is retained in the synthetic protein MFHR1, we compared mossMFHR1 to FH for their cofactor activity in a fluid-phase assay. We used mossFH because mossFH and plasmaFH exhibited comparable cofactor activities before (Michelfelder et al., 2017). Moreover, we preferred mossFH for its homogeneity. In contrast to the recombinant mossFH, plasma-purified FH derives from several donors, thus being a mixture of FH variants with polymorphisms in some positions. The cleavage of C3b is indicated by a decrease of the C3b α’-chain and occurrence of the cleavage products α’68-, α’46-, and α’43. Cofactor activity was analyzed by SDS-PAGE and Coomassie staining (Figure 5A), and the intensity of α’-chain bands was quantified by densitometry (Figure 5B). In a dose-dependent manner, mossMFHR1 and mossFH contributed to the FI-mediated degradation of the C3b α’-chain into α’68-, α’46-, and α’43 kDa fragments while the β-chain remained unchanged. Hence, MFHR1 showed comparable cofactor activity to FH.

### MossMFHR1 Accelerates the Decay of the C3 Convertase and Inhibits Further Alternative Complement Pathway

**Figure 6:**
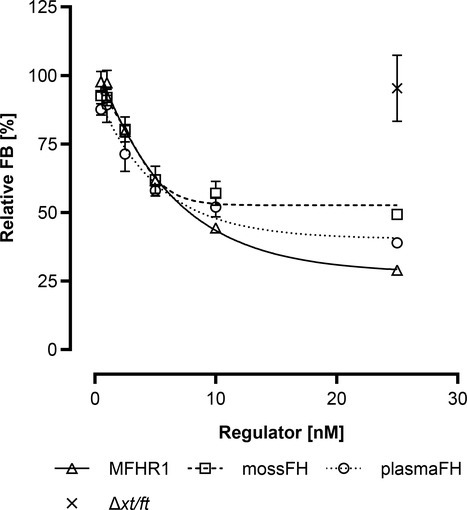
MossMFHR1 displays decay acceleration activity on the C3 convertase C3bBb. Like mossFH and plasmaFH, mossMFHR1 accelerated the decay of Bb from C3 convertase C3bBb complex in a dose-dependent manner. The OD at 450 nm (OD_450_) of preformed C3bBb (C3b + FB + FD) without any regulator was set to 100%. C3bBB OD_450_ (only C3b + FB without any FD and regulator) was set to 0%. Purified extract of the parental moss strain *Δxt/ft* was included as a control, in equal amounts to the volume of MFHR1 used for the highest concentration. Data represent mean values and ± SD from three replicates and were analyzed with two-way ANOVA followed by Bonferroni test. Some error bars are shorter than the symbol. Analyses were done with GraphPad Prism software version 8.0 for Windows.

The so-called decay acceleration activity of mossMFHR1 was compared with plasma-derived FH (plasmaFH) and mossFH *in vitro*. C3b together with FB generates a complex that is cleaved by FD to generate the active C3 convertase C3bBb, which promotes the alternative pathway, intensifying the reaction *via* the amplification loop (Figure 1A). On the contrary, FH reduces the level of active C3 convertase C3bBb by removing the Bb portion, thus inactivating the AP (Harris et al., 2005). As expected, mossMFHR1 accelerated the decay of the C3 convertase C3bBb and even performed significantly better than plasmaFH and mossFH at higher concentrations of 10 and 25 nM (*p* < 0.05) (Figure 6).

### MossMFHR1 Inhibits the AP Activation in Human Blood

**Figure 7:**
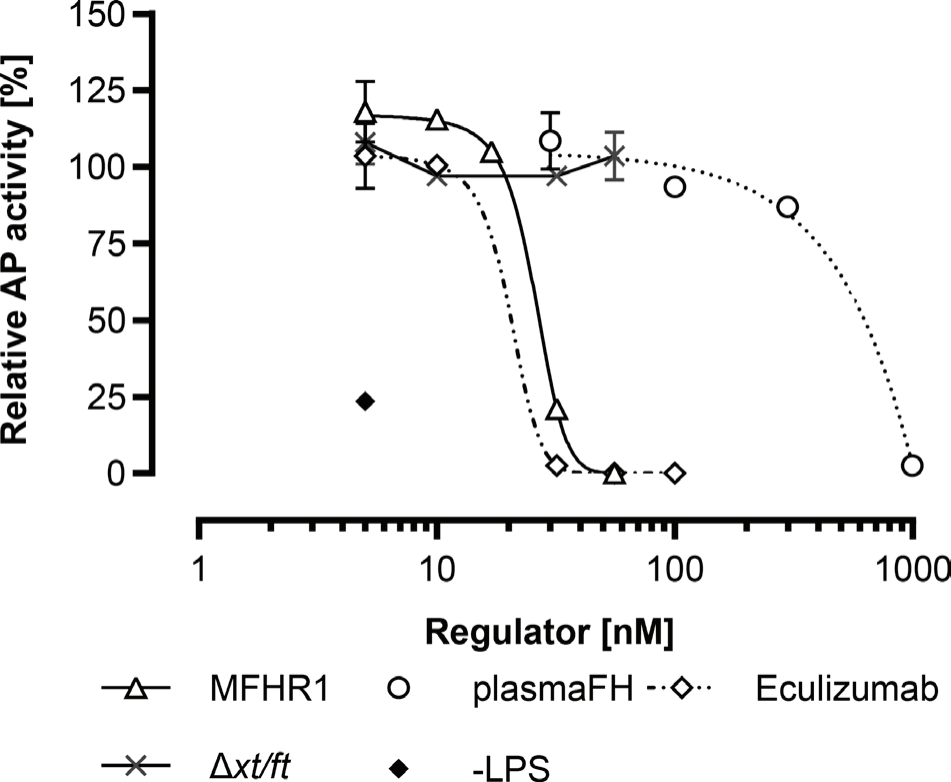
MossMFHR1 inhibits the AP activity after induction of the alternative pathway in human serum. The C5b-9 complex formation was measured by ELISA after LPS-induced AP activation. Data represent mean values and bars show the range of duplicates and were analyzed with two-way ANOVA followed by Bonferroni test. The serum without regulators was set to 100% and heat-inactivated serum to 0%. Activity in wells not coated with LPS (-LPS) was used as a reference. Purified extract of the parental moss strain *Δxt/ft* was included as a negative control, in equal amounts to the volume of MFHR1 used. Analyses were done with GraphPad Prism software version 8.0 for Windows.

To check the additional regulatory capacity of MFHR1 which derives from FHR1 domains, we measured the ability of mossMFHR1 to inhibit the formation of C5b-9, i.e. the terminal complement complex (TCC), after activation of the cascade with bacterial lipopolysaccharide (LPS) in human blood serum. In this assay, the regulatory activities on all AP levels are evaluated together. Increasing amounts of mossMFHR1 regulated LPS-induced AP activation and inhibited TCC formation efficiently and in most concentrations not significantly different from the therapeutic antibody Eculizumab (Figure 7); only at 32 nM did the blocking antibody perform better than mossMFHR1 (*p* < 0.05). MossMFHR1 regulated the complement cascade and the formation of the TCC much more efficiently than plasmaFH. The complete inhibition of the AP was achieved by 56 nM MFHR1; approximately, 22 times less MFHR1 was needed when compared to plasmaFH.

## Discussion

The complement system is a tightly regulated cascade that efficiently clears infectious agents and modified body cells and protects host tissues. Dysregulation of this delicately balanced cascade leads to infection and severe diseases such as atypical hemolytic uremic syndrome (aHUS), paroxysmal nocturnal hemoglobinuria (PNH), C3 glomerulopathy (C3G), age-related macular degeneration, and microangiopathic hemolytic anemia (Józsi et al., 2005). Complement factor H is a potent regulator of the alternative pathway and FH-replacement therapy could restore normal complement activity in the sera of aHUS and C3G patients (Michelfelder et al., 2017). Due to its high molecular mass and biochemical complexity, recombinant production of FH is far from trivial, thus no recombinant product is under clinical evaluation (Yang et al., 2018). Recently, mossFH showed full *in vitro* complement regulatory activity and decreased deposition of C3 cleavage products in preclinical studies in a murine model of C3G, indicating improved kidney function (Michelfelder et al., 2017). The therapy with Eculizumab, which binds C5 and inhibits its activation, improved disease progression, survival, and quality of life in patients with aHUS and PNH but showed partial response only in some patients suffering from C3G, most likely due to the uncontrolled over-activation of the cascade in steps previous to Eculizumab’s point of action (Bomback et al., 2012). Thus, recombinant proteins modulating the complement system on multiple levels might be key to the development of new therapeutics to treat complement-related disorders. MFHR1, a novel synthetic multitarget complement inhibitor designed to combine the terminal pathway regulatory activity of FHR1 with the regulatory domains for C3 level control and binding affinity to host cell surfaces of FH, was synthesized in a proof-of-concept study with transiently transfected insect cells and shown to be a promising multilevel complement regulator (Michelfelder et al., 2018). This study demonstrated that MFHR1 can achieve the same complement regulatory activity as plasmaFH at much lower concentrations. Being FH a very abundant serum protein with concentrations of approximately 500 μg/ml, the amount of protein needed per patient is important for the feasibility of the treatment. Baculovirus-infected insect cells are used for heterologous protein expression widely for two reasons: availability of two strong promoters which drive target protein expression to high levels (Scholz and Suppmann, 2017) and lepidopteran cell lines that can perform post-translational modifications and grow to high cell densities (Berger et al., 2004; Van Oers et al., 2015). On the other hand, there are several drawbacks including demanding and expensive culture conditions (Gecchele et al., 2015), batch-to-batch inconsistency due to the lot-to-lot variability of media (Chan and Reid, 2016), the need of large volumes of viruses to scale up protein production, different glycosylation pattern (paucimannosidic N-glycans) than humans (Shi and Jarvis, 2007), potential cell lysis, and degradation of protein of interest caused by viral infection (Broadway, 2012). Therefore, after demonstrating the bioactivity of insect-cell expressed MFHR1 protein, we now aimed to establish a stable production process for MFHR1 in the moss bioreactor. This system proved its validity as a biopharmaceutical production host by successfully completing the phase I clinical trial for moss-aGal (Reski et al., 2018). Moreover, it was the first system to succeed in the stable production of active recombinant human FH (Büttner-Mainik et al., 2011; Michelfelder et al., 2017).

After the characterization of transgenic moss lines in terms of growth performance and MFHR1 production levels in bioreactors, the line P1 was found to be most promising with production levels of 100 μg MFHR1/g FW and a 54-time increment of the biomass within 9 days of cultivation in a 5-L bioreactor.

The endogenous actin 5 promoter, and the 5’ UTR including its intron, is a valuable tool for the production of pharmaceutically interesting proteins in *P. patens* (Baur et al., 2005a,b; Weise et al., 2006, 2007; Büttner-Mainik et al., 2011; Michelfelder et al., 2017). For proteins expressed under the control of this promoter, a temporal increase in the production of the protein of interest in *P. patens* could be achieved by the addition of auxin (data not shown). This effect could be shown here as the addition of the synthetic auxin NAA increased MFHR1 levels. Auxin also stimulated the ACT7-GUS reporter in *Arabidopsis thaliana* (Kandasamy et al., 2001). To attain an increase in recombinant protein yield, however, auxin concentration and time of exposition had to be adapted to each protein of interest and culture conditions.

According to mass spectrometry, mossMFHR1 is completely and correctly synthesized with a moss-derived signal peptide (Schaaf et al., 2004) and properly processed as demonstrated before for moss-produced recombinant human VEGF and FH (Gitzinger et al., 2009; Büttner-Mainik et al., 2011; Michelfelder et al., 2017). In addition, complex-type N-glycosylation of mossMFHR1 was confirmed by MS-based glycopeptide analysis. The site NGS originated from FH SCR_4_ was not glycosylated, but deamidated to DGS, as observed before for both, human plasma-purified as well as moss-produced FH (Fenaille et al., 2007; Michelfelder et al., 2017). The other MFHR1 N-glycan site, located in FHR1-derived SCR2, was occupied in approximately 35% of the molecules predominantly with GnGn N-glycans. A small portion harbored monoantennary Lewis A structures. The moss α1,4 fucosyltransferase and β1,3 galactosyltransferase responsible for this modification have already been identified and could be further eliminated by knocking out the responsible genes as it was already achieved for asialo-EPO production in moss (Parsons et al., 2012). N-glycans with terminal mannoses were less than 1% and as expected from the glyco-engineered *Δxt/ft* parental line, putatively immunogenic β1,2-linked xylose and α1,3-linked fucose never appeared. The fact that the majority of mossMFHR1 molecules was not glycosylated is in full agreement with the situation described for human FHR1 (Skerka and Zipfel, 2008). Considering the different aspects of glycosylation, the mossMFHR1 presented here provides optimal conditions for a safe, non-immunogenic pharmaceutical.

Having proven its structural integrity, the different proposed functions of mossMFHR1 were subsequently tested. Aiming at a multilevel activity from combining the relevant functional domains of FHR1 and FH, we checked both, the functions of FH domains, by controlling the early stages of AP activation (proximal part) in the fluid phase and of FHR1 in the terminal part, blocking TCC formation on target structures. FH is inhibiting the activation of the complement cascade on two levels: as a cofactor for FI-mediated cleavage of C3b and in accelerating the decay of the crucial enzyme of complement activation, the C3 convertase. Both functions were executed by mossMFHR1 in a similar or even more efficient way compared to the full-length mossFH (Michelfelder et al., 2017) or human plasma-purified FH. MossMFHR1 degraded the C3 convertase like plasmaFH and mossFH at low concentrations and performed significantly better than both FH versions at higher concentrations. MFHR1 activity for inhibiting the terminal pathway was measured as decrease of C5b-9 (TCC) formation and compared to the activity of the C5-inhibiting antibody Eculizumab as well as plasmaFH. MossMFHR1 inhibited TCC formation in a similar manner as Eculizumab and 22 times more efficiently than plasmaFH. These results are in accordance with those obtained with the insect cell-derived MFHR1 (Michelfelder et al., 2018) and a similar fusion protein published recently (Yang et al., 2018). The improved activity compared to native FH might be explained not only by the combined activity at different levels of the alternative pathway but also by the formation of MFHR1 homodimers deriving from the FHR1 dimerization domain included in MFHR1 (Michelfelder et al., 2018; Yang et al., 2018).

These encouraging results, the structural integrity and increased activity combined with a smaller molecule size, strongly recommend the initiation of large-scale cultivation for proving the mossMFHR1’s *in vivo* therapeutic activity. In summary, mossMFHR1 showed its high potential to become a new and indispensable drug for patients with complement-associated disorders.

## Materials and Methods

### Construct Generation

For efficient production of MFHR1, the vector pAct5-MFHR1 was used, where the expression of the MFHR1 coding sequence is driven by the 5′ region, including the 5′ intron, of the PpActin5 gene (Weise et al., 2006). The CaMV 35S terminator preceded by an 8x His-tag and a SalI restriction site was amplified from the pRT_VEGF_121_ plasmid (Koprivova et al., 2004). In addition, this plasmid includes an hpt cassette for selection of transformed plants with hygromycin (Supplementary Figure 5).

The targeting of the mossMFHR1 to the secretory pathway for proper posttranslational modifications was driven by the aspartic proteinase signal peptide from *P. patens*, PpAP1 (Schaaf et al., 2004, 2005). Its coding sequence was amplified from the plasmid pFH (Büttner-Mainik et al., 2011) with the primers XhoI_MFHR1_F (5′-TCTCGAGATGGGGGCATCGAGG-3′) and AP_MFHR1_R (5′-ATCACAAAATGTTGCTTCTGCCTCAGCTAAGGC-3′). The coding sequence for the MFHR1 was amplified from pFastbac-MFHR1 (Michelfelder et al., 2018) with the primers AP_MFHR1_F (5′-GAGGCAGAAGCAACATTTTGTGATTTTCCAAAAATAAACC-3′) and SalI_MFHR1_R (5′-TGTCGACTCTTTTTGCACAAGTTGGATACTC-3′). The signal peptide and the MFHR coding sequence were assembled *via* two-template PCR with the primers XhoI_MFHR1_F and SalI_MFHR1_R and cloned into the expression vector *via* XhoI and SalI restriction sites.

All amplifications were performed by phusion DNA polymerase (Thermo Fisher Scientific, Waltham, MA, USA)-based PCR.

### Plant Material and Cell Culture

*Physcomitrella patens* (Hedw.) Bruch & Schimp was cultivated as described previously (Frank et al., 2005). The MFHR1-producing moss lines were obtained by stable transformation of the Δ*xt/ft* moss line, in which the α1,3 fucosyltransferase and the β1,2 xylosyltransferase genes have been disrupted *via* homologous recombination. Transfection was performed with 40 μg of linearized MFHR1 construct per reaction as described before (Decker et al., 2015). Subsequently, selection of stable transformants on solidified Knop medium containing 25 mg/L hygromycin was performed as described previously (Decker et al., 2015).

Plants surviving the selection with hygromycin were screened for the presence of the transgene in the moss genome by PCR, as described before (Parsons et al., 2012), using the primers MFHR1_fwd (5′-GAAGGATGGTCACCAACACC-3′) and MFHR1_rev (5′-CATTGGTCCATCCATCTGTG-3′). The production of the protein of interest was tested *via* ELISA. For this purpose, approximately 10 mg of plant material were picked from colonies growing on Knop solid medium and transferred to 2-ml tubes with one tungsten carbide (Qiagen, Hilden, Germany) and one glass (Roth, Karlsruhe, Germany) beads, diameter 3 mm. After the addition of 120 μl extraction buffer (408 mM NaCl, 60 mM Na_2_HPO_4_, 10.56 mM KH_2_PO_4_, 60 mM EDTA, pH 7.4 and 1% protease inhibitor (P9599, Sigma-Aldrich), plant material was homogenized for 1 min by the use of a mixer mill (MM 400, Retsch, Haan, Germany) at 30 Hz and subsequently sonicated for 10 min in a ultrasound bath (Bandelin Sonorex RK52). Extracts were analyzed *via* ELISA as described before (Büttner-Mainik et al., 2011), but using the anti-FH antibody GAU 018-03-02 (Thermo Fisher Scientific) as capture antibody 1:2,000 in coating buffer, and using FH as standard protein (Calbiochem, San Diego, CA, USA) for the screening of MFHR1-producing plants. Under these conditions, the ELISA has a linear response from 1 to 64 ng/ml. For the quantification of MFHR1 produced by plants grown in liquid media, the protein extraction was performed as described above, but the plant material was first vacuum-filtrated, frozen, and disrupted frozen before the addition of the buffer.

For production of MFHR1, the P1 line was cultivated at pH 4.5 in a 5 L photobioreactor as described previously (Hohe and Reski, 2005), with continuous light at 350 μE intensity. Daily from day 6 to day 8, 2 L suspensions were harvested and replaced by fresh medium. Plant material was vacuum-filtrated and shock-frozen in N_2_, and stored at −80°C until further processing. At day 8, after harvesting, 250 μl 100 mM naphthaleneacetic acid (NAA, Sigma) was added to the bioreactor to reach a final concentration of 5 μM NAA. At day 9, the whole culture was harvested.

### Purification of Recombinant MFHR1

Frozen plant material was resuspended in binding buffer (0.75 M NaCl, 75 mM Na_2_HPO_4_, 10 mM Imidazole, 1% protease inhibitor, pH 8.0) in a ratio plant material:buffer 1:4, and homogenized for 10 min with an Ultra-Turrax at 10,000 rpm on ice. After centrifugation, the supernatant was filtrated through 1 μm polyethersulfon (PES) filters (Whatman, GE Healthcare UK Limited, Buckinghamshire, UK) and subsequently through 0.22 μm PES filters (Roth).

Chromatography was performed using an ÄKTA system (GE Healthcare, Uppsala, Sweden) at a flow rate of 1 ml/min. The filtered cellular extract was applied to a 1 ml (CV) HisTrap FF column (GE-Healthcare). After washing with 30 CV binding buffer supplemented with 3% buffer B (500 mM NaCl; 500 mM imidazole; 100 mM Na_2_HPO_4_; pH 8.0), elution was performed in a gradient of 3–100% of buffer B in 9 CV and recovered in 0.5 ml fractions. Fractions containing the protein of interest with a negligible amount of contaminant proteins were pooled (fractions 10–16), dialyzed against DPBS (Gibco® by Life Technologies, Darmstadt, Germany) in two Slide-A-Lyzer® MINI Dialysis Devices, 20 K MWCO and concentrated using Vivaspin membrane filter devices (Sartorius AG, Goettingen, Germany) with a 10 kD MWCO. Subsequently, the product was aliquoted, shock frozen in liquid N_2_, and stored at −80°C until further analysis.

### Protein Quantification

The concentration of mossMFHR1 used for activity assays was measured *via* ELISA, using the same antibodies and protocol performed for mossFH as described above. As standard for the ELISA, a batch of purified mossMFHR1 was used in which its concentration was determined *via* band densitometry (Quantity One, Bio-Rad, Munich, Germany) after SDS–PAGE (Ready Gel Tris-HCl, Bio-Rad) and Coomassie staining. The sandwich ELISA using this MFHR1 standard protein has a linear response between 1 and 64 ng/ml. Electrophoretic separation of proteins was carried out in 10% SDS–polyacrylamide gels (Ready Gel Tris-HCl; BioRad) at 120 V. Subsequently, gels were stained with PageBlue® Protein Staining Solution (PageBlue™, Thermo Fisher Scientific). For Western blot analysis, SDS-PAGE gel was blotted to polyvinylidene fluoride (PVDF) membranes (Immobilon-P; Millipore, Bedford, MA, USA) in a Trans-Blot SD Semi-Dry Electrophoretic Cell (Bio-Rad) for 1 h with 1 mA /cm^2^ membrane. Immunoblotting was performed using mouse anti-His antibodies (MAB050, R&D Systems, Minneapolis, MN, USA) as primary and sheep anti-mouse antibodies coupled to peroxidase (NA931, Amersham ECL™, GE Healthcare) as secondary antibody in a 1:500 and 1:10,000 dilution respectively, followed by chemiluminescence development (ECL™ Advance Western Blotting Detection Kit, GE Healthcare) following the manufacturer’s instructions.

### Glycopeptide Analysis

Glycopeptide analysis was performed from samples purified *via* Ni-NTA. These were mixed 1:1 with 2x sample loading buffer (Bio-Rad) with 50 mM DTT, incubated for 5 min at 95°C and after cooling down to room temperature the samples were S-alkylated. Electrophoretic separation and Coomassie staining was performed as described above, and the bands corresponding to mossMFHR1 (monitored by a parallel Western blot) were cut and digested with trypsin overnight. Tryptic peptides were extracted first with 100% acetonitrile (ACN) followed by 5% formic acid and glycopeptide enrichment procedure was modified after (Kolarich et al., 2012) using HILIC HyperSep™ Tips (Thermo Fisher Scientific). Gel extracts were dried in a vacuum concentrator and dissolved in 100 μl HILIC binding buffer (85% Acetonitrile, 15 mM ammonium acetate, pH 3.5). Each HILIC tip was equilibrated with 20 μl binding buffer. About 100 μl sample was loaded by up and down pipetting for 40 times. The flow-through was collected and dried in a vacuum concentrator. The loaded HILIC tip was washed with 20 μl binding buffer by up and down pipetting for 20 times. Glycopeptides were eluted in 20 μl 15 mM ammonium acetate (pH 3.5) and dried by vacuum concentration. The dried flow-through fraction was dissolved in 100 μl 0.1% formic acid and desalted using C18 StageTips (Thermo Fisher Scientific). C18 Tips were equilibrated successively with 100 μl 0.1% formic acid, next with 100 μl 80% ACN with 0.1% formic acid and finally again with 100 μl 0.1% formic acid. The dissolved flow-through was loaded, washed with 100 μl 0.1% formic acid, and eluted in 100 μl ACN with 0.1% formic acid. The elution fraction was dried in a vacuum concentrator. Both the HILIC and the C18 eluates were dissolved in 0.1% formic acid and measured using the UltiMate 3,000 RSLCnano system (Dionex LC Packings/Thermo Fisher Scientific, Dreieich, Germany) coupled online to a QExactive Plus instrument (Thermo Fisher Scientific, Bremen, Germany). For the UHPLC systems, a C18-precolumn (Ø 0.3 mm, length 5 mm; PepMap, Thermo Fisher Scientific) and an Acclaim® PepMap analytical column (*ID*: 75 μm, 500 mm, length 2 m, 100 Å, Dionex LC Packings/Thermo Fisher Scientific) were used. Washing and pre-concentration of samples took place on a C18-precolumn with 0.1% formic acid (solvent A) for 5 min before peptides entering the analytical column. With a flow rate of 250 nl/min, peptide separation was performed applying a 45-min gradient of 4–40% solvent B (0.1% formic acid/86% acetonitrile) in 30 min and 40–95% solvent B in 5 min. After each gradient, the analytical column was washed with 95% solvent B for 5 min and re-equilibrated for 15 min with 4% solvent B. MS/MS analyses were performed on multiply charged peptide ions. To automatically switch between MS (max. of 1 × 10 ions) and MS/MS the instrument operation took place in the data-dependent mode. After MS scan, a maximum of 12 precursors were selected for MS/MS scans using HCD with normalized collision energy of 35%. The mass range for MS was m/z = 375–1,700 and resolution was set to 70,000. MS parameters were as follows: spray voltage 1.5 kV and ion transfer tube temperature 200°C. Raw data analysis was performed using Mascot Distiller V2.5.1.0 (Matrix Science, USA), and the peak lists were searched with Mascot V2.6.0 against an in-house database containing all *P. patens* V1.6 protein models (Zimmer et al., 2013) as well as the MFHR1 sequence.

For database searching, the following parameters were used: peptide mass tolerance: 5 ppm, MS/MS mass tolerance: 0.02 Da, enzyme: trypsin with maximum two missed cleavages, variable modifications: Gln− > to pyroGlu (N-term. Q) −17.026549 Da, oxidation (M) and carbamidomethyl (C). +57.021464 Da, Hydroxyproline (P) +15.994915 Da. Quantitation of peptides was done with Excalibur Qual Browser V2.2.44 (Thermo Fisher Scientific) employing manual peak area integration of extracted ion chromatograms. For all glycopeptides identified, the presence of the GlcNAc-specific reporter ions (Halim et al., 2014) was inspected in the corresponding MS/MS spectra to confirm the presence of glycan structures. The following m/z-values were expected: LQNNENNISCVER [M + 2H^+^]: 795.3704, LQNNENNISCVER_GnGn [M + 3H^+^]: 963.4081, LQNNENNISCVER_GnAF [M + 3H^+^]: 1066.1116.

### Cofactor Activity

C3b proteolytic degradation regulatory activity of mossMFHR1 and plasmaFH was compared in a fluid phase cofactor assay modified from Michelfelder et al. (2017). Briefly, in a 20 μl reaction, increasing doses of mossMFHR1 or plasmaFH, and corresponding maximal volume of the parental plant Δ*xt/ft* purified extract as control, were incubated with 2 μg C3b and 500 ng complement factor I (FI) (CompTech, Texas, USA) in PBS at 37°C for 30 min. The reaction was stopped by the addition of sample loading buffer (Bio-Rad) under reducing conditions (50 mM DTT, NuPAGE™, Thermo Fisher Scientific). The FH and FI catalyzed proteolytic cleavage of C3b into iC3b was analyzed by visualizing the α-chain cleavage fragments α’68 and α’43 by SDS-Page in 10% SDS–polyacrylamide gels and Coomassie staining. The remaining intact α-chain was quantified by band densitometry (Quantity One, Bio-Rad).

### Decay Acceleration Activity

The ability of mossMFHR1 to displace the fragment Bb from the preformed C3 convertase complex C3bBb was measured by ELISA as described previously (Michelfelder et al., 2017). Purified C3b (CompTech) was immobilized on Maxisorb plates and 400 ng Factor B and 25 ng Factor D (CompTech) in phosphate buffer (containing 2 mM NiCl_2_, 25 mM NaCl, and 4% BSA) was added to the wells and incubated for 2 h at 37°C to generate C3bBb complex. Various doses of MFHR1, mossFH, plasmaFH, or Δ*xt/ft* were added and incubated for 30 min at 37°C. Afterward, intact C3bBb complexes were measured by FB-specific antibody (1:2,000; Merck, Darmstadt, Germany), followed by HRP-conjugated rabbit anti-goat (1:5,000; Dako, Hamburg, Germany). The OD at 450 nm (OD_450_) was obtained after the incubation with TMB Substrate. The OD_450_ of preformed C3bBb (C3b + FB + FD) without any regulator was set to 100%. C3bBB OD_450_ (only C3b + FB without any FD and regulator) was set to 0%.

### Determination of AP-Activity in Human Serum

The ability of mossMFHR1 to inhibit the AP activity in normal human serum (NHS) was determined by measuring the Terminal Complement Complex formation inhibition *via* ELISA as previously described (Michelfelder et al., 2018). In this ELISA-based method, the amount of active C5b-9 formation is measured during incubation of serum in wells pre-coated with lipopolysaccharide (LPS) in the presence of AP-pathway-specific conditions. The amount of bound C5b-9, detected *via* specific antibodies, is directly proportional to the activity of the AP. Extracts from the parental plant Δ*xt/ft* were used as negative controls. The OD_450_ obtained for samples with heat-inactivated serum instead of NHS were set to 0% and samples without any addition of regulators were set to 100% of C5b-9 formation.

## Statistical Analysis

Analyses were done with the GraphPad Prism software version 8.0 for Windows (GraphPad software, San Diego, California, USA; www.graphpad.com).

## Data Availability

All datasets generated for this study are included in the manuscript and/or the supplementary files.

## Author Contributions

OT and JP purified the protein and performed the activity tests. JP carried out the moss cultures in the bioreactor. LB and SH performed the mass spectrometric analysis. PK cloned the expressing vector and transformed *P. patens* with it. CB-S screened the putative producing lines. SM cloned the coding sequence and set up the activity test protocols. OT, JP, KH, PZ, RR and ED designed the study and wrote the manuscript.

### Conflict of Interest Statement

The authors are inventors of patents and patent applications related to the production of recombinant proteins in *P. patens*. RR is an inventor of the moss bioreactor and a founder of Greenovation Biotech. He currently serves as advisory board member of this company.

## Footnotes

^1^http://medicago.com

^2^https://www.greenovation.com
