# Supplementary material for: Recombinant Production of MFHR1, A Novel Synthetic Multitarget Complement Inhibitor, in Moss Bioreactors
Source: Front Plant Sci. 2019 Mar 20;10:260. doi: 10.3389/fpls.2019.00260 (PMC6436476; doi:10.3389/fpls.2019.00260)
Supplement: Supplementary file 1 [file Data_Sheet_1.docx]

# SUPPLEMENTARY material


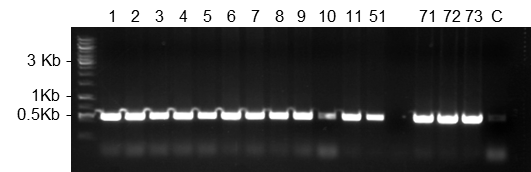


**Supplementary Figure 1.** PCR based screening for the presence of the transgene in the genome of moss transgenic lines surviving after selection. The expected size of the PCR product was 559 bp. C: parental plant Δ*xt/ft*.

**Supplementary Table 1.** Screening of promising plants grown in liquid culture for productivity via ELISA

| Lines | µg MFHR1/g FW |
| --- | --- |
| P1 | 14.0 |
| P4 | <0.1 |
| P5 | 12.5 |
| P101 | 1.1 |
| P102 | 0.1 |
| P105 | 0.1 |
| HRP 1 | 6.6 |
| HRP 15 | 3.7 |
| HRP 27 | 1.3 |

**
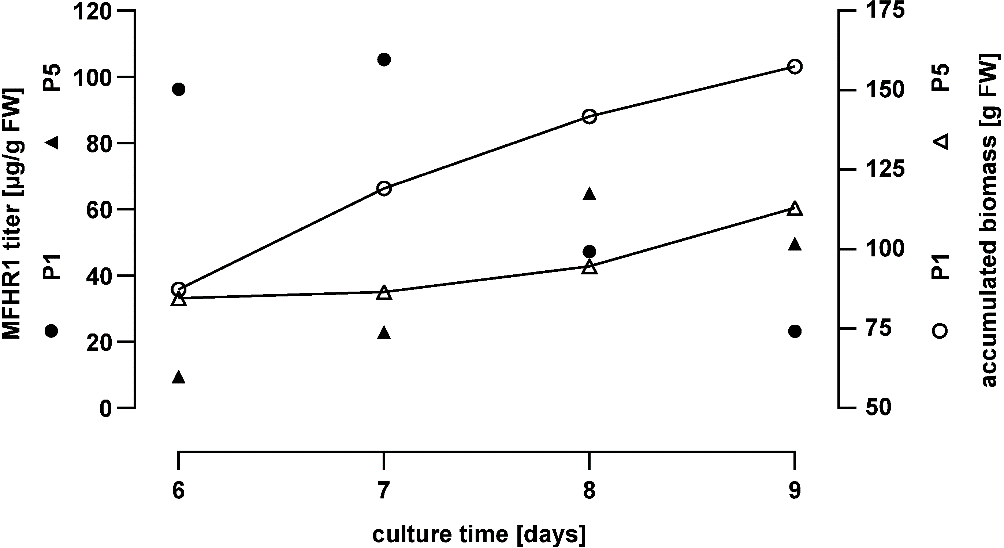
**

**Supplementary Figure 2.** Kinetics of biomass accumulation and MFHR1 concentration of lines P1 and P5 in batch cultures carried out in a 5 L photobioreactor.

**
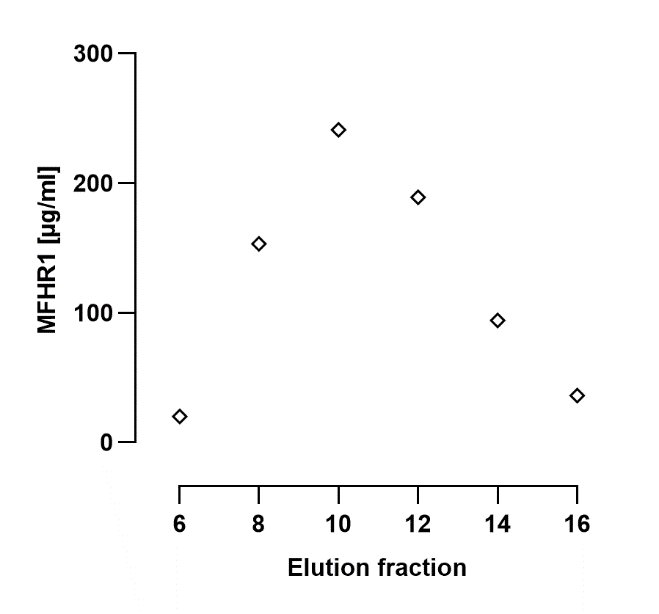
**

**Supplementary Figure 3.** MFHR1 concentration of every second elution fraction from the Ni-NTA affinity chromatography, from 6^th^ to 16^th^.


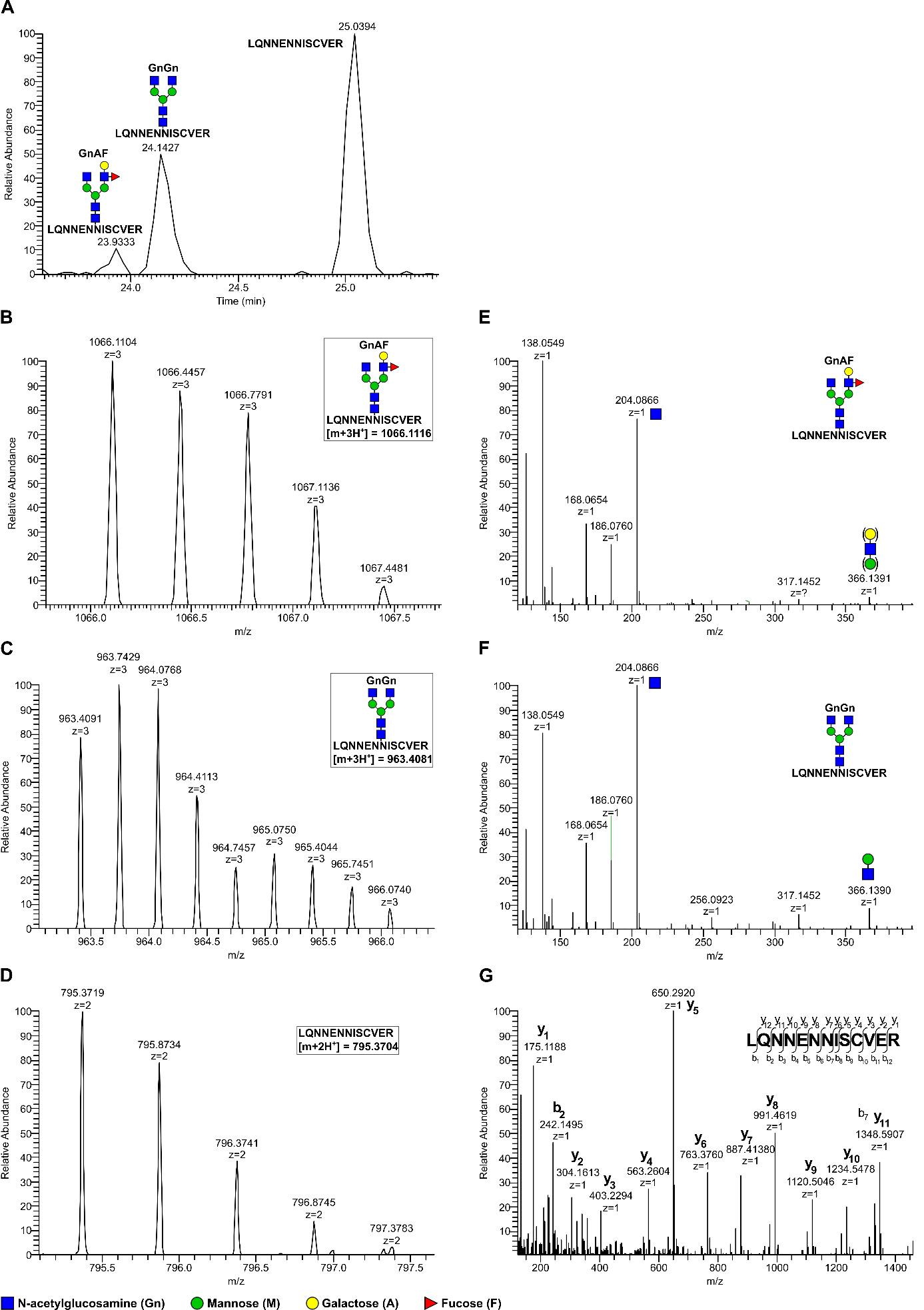


**Supplementary Figure 4.** Confirmation of peptide and glycopeptide identity flanking Asn108 on MS1-level and fragment ion spectra (MS2). (A) Repetition of Figure 4B for better clarity – showing the elution profiles (extracted ion chromatogram, EIC) of the tryptic peptide (_102_LQNNENNISCVER_113_) which flanks the glycosylation site NIS (Asn108) with and without N-glycosylation. (B,C,D) Isotope patterns of identified peptides and glycopeptides, resp., on MS1 level showing observed charge states and m/z ratios (MS1 tolerance: 5 ppm). (E,F,G) Fragment ion spectra (MS2) corresponding to the selected precursors (left panel (B,C,D). Confirmation of glycopeptide identity (E,F) was done by the presence of reporter ions from glycan structures such as N-acetylglucosamine (Gn, m+H^+^: 204.0872) and its typical oxonium ion series (Gn-H_2_O, m+H^+^: 186.0766; Gn-2H_2_O, m+H^+^: 168.0661; Gn-CH_6_O_3,_ m+H^+^: 138.0555) as well as N-acetylglucosamin-Hexose (Gn-Hex, m+H^+^: 366.1395). (G) Fragment ion spectrum of the unglycosylated peptide flanking Asn108 with identified Y-ion series. M: Mannose Gn: N-acetylglucosamine, A: Galactose, F: Fucose.
